# Sazetidine-A Activates and Desensitizes Native α7 Nicotinic Acetylcholine Receptors

**DOI:** 10.1007/s11064-014-1302-6

**Published:** 2014-04-12

**Authors:** Jack L. Brown, Susan Wonnacott

**Affiliations:** Department of Biology and Biochemistry, University of Bath, Bath, BA2 7AY UK

**Keywords:** Live cell calcium imaging, SH-SY5Y cells, Primary cortical neurons, PNU-120596

## Abstract

The aim of this study was to investigate the ability of sazetidine-A, a novel partial agonist at α4β2 nicotinic acetylcholine receptors (nAChRs), to affect the function of native α7 nAChRs in SH-SY5Y cells and primary cortical cultures. The α7-selective positive allosteric modulator PNU-120596 was used to reveal receptor activation, measured as an increase in intracellular calcium using fluorescent indicators. In the absence of PNU-120596, sazetidine-A elicited mecamylamine-sensitive increases in fluorescence in SH-SY5Y cells (EC_50_ 4.2 µM) but no responses from primary cortical neurons. In the presence on PNU-120596, an additional response to sazetidine-A was observed in SH-SY5Y cells (EC_50_ 0.4 µM) and robust responses were recorded in 14 % of cortical neurons. These PNU-120596-dependent responses were blocked by methyllycaconitine, consistent with the activation of α7 nAChRs. Preincubtion with sazetidine-A concentration-dependently attenuated subsequent responses to the α7-selective agonist PNU-282987 in SH-SY5Y cells (IC_50_ 476 nM) and cortical cultures. These findings support the ability of sazetidine-A to interact with α7 nAChRs, which may contribute to sazetidine-A’s actions in complex physiological systems.

Sazetidine-A (6-[5-[(2S)-2-azetidinylmethoxy]-3-pyridinyl]-5-hexyn-1-ol) has the profile of a potent partial agonist at α4β2 nicotinic acetylcholine receptors (nAChRs) and has attracted interest as a lead compound for several therapeutic targets, making detailed knowledge of its wider activity an important consideration. Sazetidine-A is a derivative of the nicotinic agonist A-85380, developed to bear a long side chain for potential attachment of fluorescent or photoaffinity probes [1]. In binding assays it showed improved selectivity for α4β2 nAChRs, compared with A-85380, with K_i_ values of 0.4 and 0.6 nM for rat and human α4β2 nAChRs respectively.

In contrast to the parent molecule, sazetidine-A appeared to be devoid of agonist activity at recombinant human α4β2 nAChRs and was described as a ‘silent desensitizer’ [1]. Subsequent studies revealed sazetidine-A to be a stoichiometry-dependent agonist capable of fully activating high sensitivity human α4_2_β2_3_ (HS-α4_2_β2_3_) nAChRs, whereas it had negligible efficacy, relative to acetylcholine, at lower sensitivity α4_3_β2_2_ (LS-α4_3_β2_2_) nAChRs [2, 3]. Presumably the stable cell lines employed in the original study predominantly expressed the low sensitivity form. The ‘accessory’ subunit occupying the fifth position in the pentameric nAChR, a position that does not directly contribute to either of the two high affinity, orthosteric agonist binding sites, nevertheless can influence receptor properties [4]. In this case the subunit occupying this position appears to determine the ability of sazetidine-A to affect the opening of the α4β2 nAChR channel. This notion is reinforced by the opposing effects of α5 and β3 subunits in the fifth position: inclusion of an α5 subunit to form α4_2_β2_2_α5 nAChRs abolished agonist efficacy [5], whereas sazetidine-A is a potent and efficacious agonist at α4α6β2_2_β3 nAChRs (EC_50_ 19 nM) [6].

The sobriquet ‘silent desensitizer’ reflected the observation that a brief preincubation with 1 µM sazetidine-A induced a long lasting inhibition of recombinant human α4β2 nAChRs expressed in SH-EP1 cells (IC_50_ value ~ 30 nM), compared with the rapid recovery following preincubation with nicotine [1]. More recent studies that have taken into account its differential interaction with the two stoichiometries of α4β2 nAChRs have shown that sazetidine-A selectively desensitizes HS-α4_2_β2_3_ nAChRs over LS-α4_3_β2_2_ [7, 8].

Its partial agonist profile has raised interest in sazetidine-A as a therapeutic lead for numerous indications, including drug dependence [9, 10], cognitive and attentional deficits [11], pain [12] and depression [13]. Given the complex contributions of multiple nAChRs in the CNS to brain function and behaviours, it is important to understand the specificty of agents such as sazetidine-A. It binds with much lower affinity but acts as a full or substantial agonist at recombinant α4β4, α3β4, and α6β2*nAChRs [1, 6, 7]. Sazetidine-A’s activation of α7 nAChRs has only recently been documented, with very different EC_50_ values and efficacies at human (1.2 µM; 100 %) and rat (60 µM; 6 %) α7 nAChRs [7, 14]. The contribution of α7 nAChRs to the clinical targets mentioned above [15, 16], as well as α7 nAChRs credited with functions in peripheral systems that could mediate side-efffects [17] suggest that this nAChR subtype, in particular, merits further attention. Hitherto, sazetidine-A’s activation of native α7 nAChRs has not been reported. In this study we examined the ability of sazetidine-A to activate and desensitize α7 nAChRs in SH-SY5Y cells and primary cortical neurons.

## Materials and Methods

### Materials

Triton X-100, (-)-nicotine hydrogen tartrate and mecamylamine hydrochloride, were purchased from Sigma-Aldrich (Poole, Dorset, UK); B27, l-glutamine, antibiotics, fluo-3 AM, fura-2 AM, and pluronic f127 were obtained from Life Technologies (Paisley, UK); sazetidine-A dihydrochloride, tetrodotoxin citrate, methyllycaconitine citrate and 5-iodo-A85380 dihydrochloride were purchased from Tocris Bioscience (Avonmouth, UK); PNU-120596 and PNU-282987 were provided by Pfizer Inc. USA; general reagents were purchased from Fisher Scientific (Loughborough, UK).

### Methods

#### SH-SY5Y Cell Culture

Human neuroblastoma SH-SY5Y cells (ECACC, Salisbury, UK; passages 16–27) were cultured as previously described [18]. In brief, cultures were maintained in Advanced Dulbecco’s modified Eagle’s media (DMEM/F12), supplemented with 2 % fetal bovine serum (FBS), 2 mM l-glutamine, 190 U/ml penicillin and 0.2 mg/ml of streptomycin in 94 × 16 mm tissue culture dishes in a humidified chamber at 37 °C with 5 % CO_2_. Cells were seeded 1:2 into 96-well plates, experiments were performed 72 h later with confluent cultures.

#### Mouse Primary Cortical Cultures

Primary cultures were prepared from embryonic mouse cortices as previously described [19]. Briefly, time-mated pregnant female CD1 mice were killed by cervical dislocation and E18 embryos were harvested. Cortices were dissected in PBS with 30 % glucose (Ca^2+^- and Mg^2+^-free) and dissociated with a fire polished glass Pasteur pipette. Tissue was centrifuged at 500 g for 5 min, resuspended in neurobasal medium supplemented with B27, 2 mM l-glutamine and 60 μg/ml penicillin and 100 μg/ml streptomycin (12 ml medium per brain). For live imaging experiments, cells were plated on 25 mm round glass coverslips (thickness no. 1) coated with 20 μg/ml poly-d-lysine, in 6-well tissue culture plates (Corning, USA). Cells were allowed to grow for 10–14 days in vitro (DIV) at 37 °C in a humidified atmosphere of 95 % air and 5 % CO_2._

### Ca^2+^ Fluorimetry

#### SH-SY5Y Cells

Increases in [Ca^2+^]_ic_ were measured as described previously [20]. Briefly, cells were washed twice with Tyrode’s salt solution (TSS: 137 mM NaCl, 2.7 mM KCl, 1.0 mM MgCl_2_, 1.8 mM CaCl_2_, 0.2 mM NaH_2_PO_4_, 12 mM NaHCO_3_, 5.5 mM glucose; pH 7.4) and incubated with the membrane-permeable, Ca^2+^ sensitive dye fluo-3 AM (10 μM) and 0.02 % pluronic F127 for 1 h at room temperature in darkness. Cells were then washed twice with TSS before pre-incubation (10 min) with 80 μl antagonists, modulators or TSS. Changes in fluorescence (excitation 485 nm, emission 538 mm) were monitored using a Fluoroskan Ascent fluorescent plate reader (Thermo Scientific, UK). Basal fluorescence was measured for 5 s before agonist (20 µl) was added and fluorescence was monitored for a further 20 s. Calibration of responses was achieved by determining the maximum and minimum fluorescence values of each fluo-3 AM signal, by application of 0.2 % Triton X-100 (F_max_) followed by 40 mM MnCl_2_ (F_min_). Data were calculated as a percentage of F_max _− F_min_. Concentration response data were fitted to the Hill equation and half maximal effective concentrations determined.

#### Cortical Cultures

Changes in [Ca^2+^]_ic_ in individual cells of mouse E18 cortical cultures grown on glass coverslips were monitored using live cell imaging (Concord System, Perkin Elmer, UK). Cortical cultures (10–14 DIV) were washed twice with calcium buffer (140 mM NaCl, 5.0 mM KCl, 1.0 mM MgCl_2_, 1.8 mM CaCl_2_, 10 mM glucose, 5.0 mM HEPES; pH 7.4) and incubated with the ratiometric Ca^2+^-sensitive dye fura-2 AM (5 μM) and 0.02 % pluronic F127 for 1.5 h at room temperature in darkness. After another two washes with buffer, coverslips were assembled into a temperature controlled (37 °C) perfusion chamber (Series 20 PH2 platform with a RC-21BR chamber, Harvard Apparatus, MA, USA) and mounted on an inverted fluorescence microscope. Buffer and drug solutions were pre-heated to 37 °C and perfused at a rate of 5 ml/min. Fura-2 AM was excited at 340 and 380 nm using a SpectroMaster I and emissions at 510 nm were detected with an intensified Ultrapix PDCI low light level CCD camera. All experiments were carried out in the presence of 1 μM tetrodotoxin (TTX) pre-incubated for at least 1 min prior to recording. During long drug pre-incubations perfusion was switched off to reduce drug use, and recording was turned off to prevent photobleaching.

Data were analysed with Ultraview software (Perkin Elmer, UK) and expressed as a ratio of F_340_:F_380_ following subtraction of background fluorescence taken from a region in which no cells could be seen. For successive drug treatments on the same cells, initial peak F_340_:F_380_ ratio for each individual responding region of interest (ROI) was normalized to 100 % following subtraction of mean basal F_340_:F_380_ ratio recorded immediately before drug application. Subsequent responses in the presence of antagonists/modulators or after washout were calculated as a percentage of the original response from the same ROI. These values were then averaged within experiments, such that n values reflect the number of independent cultures examined.

### Statistical Analysis

Statistical significance was evaluated by ANOVA with *post-hoc* test, or *t*-test as appropriate, with details given in figure legends.

## Results

### Effects of Sazetidine-A on Ca^2+^ Responses Initiated by Native Human nAChRs Expressed in SH-SY5Y Cells

**Fig. 1:**
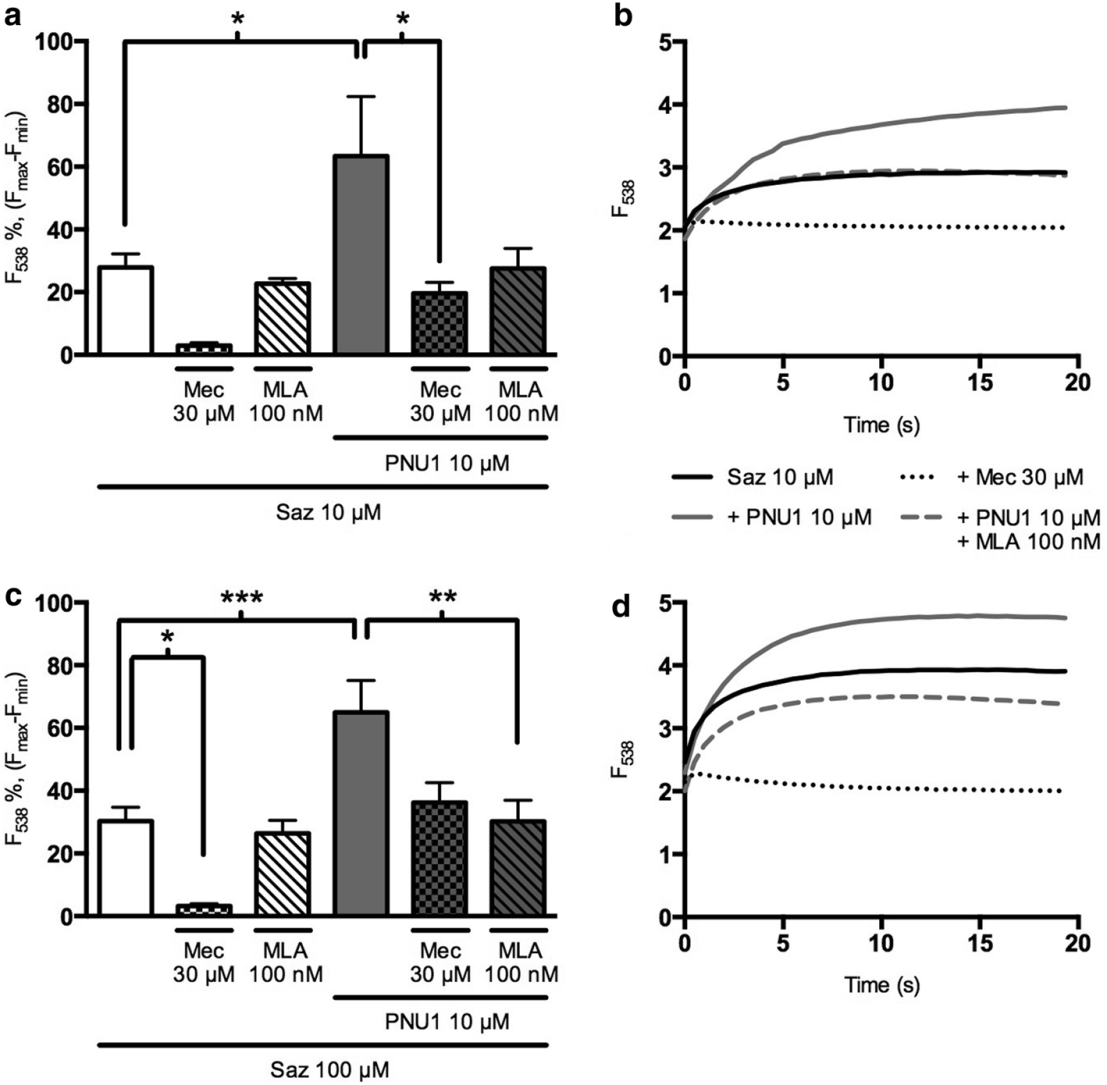
Intracellular calcium elevations evoked by sazetidine-A in SH-SY5Y cell populations. SH-SY5Y cells loaded with fluo-3 AM were preincubated for 10 min with or without antagonist (mecamylamine (Mec), 30 μM, *chequered bars*, or MLA, 100 nM, *crosshatched bars*) and/or PNU-120596 (PNU1, 10 μM, *grey bars*) before addition of sazetidine-A (Saz, 10 μΜ, **a**, **b**; 100 μM, **c**, **d**) in the presence (*grey bars*) or absence (*white bars*) of PNU-120596 (10 μM). Fluorescence at 538 nm was monitored for 20 s; representative traces are shown (**b**, **d**; some antagonist curves omitted for clarity). The increase in fluorescence at 20 s is presented as a percentage of the maximum fluorescence determined by addition of 0.2 % triton X-100 minus the minimum fluorescence quenched by 350 mM MnCl_2_ (**a**, **c**).* Bars* represent the mean ± SEM of at least 4 independent experiments; * *P* < 0.05, ** *P* < 0.01, significantly different from sazetidine-A alone or in combination with PNU-120596, non-paired one-way ANOVA, Bonferroni’s multiple comparisons test

SH-SY5Y cells express α3, α5, α7, β2, and β4 nAChR subunits [21–23] consistent with the formation of functional α3* and α7 nAChRs [20]. Based on the sensitivities of recombinant non-α4β2 nAChRs, sazetidine-A was examined at 10 and 100 µM in SH-SY5Y cells loaded with the Ca^2+^ indicator fluo-3 AM. Both concentrations of sazetidine-A produced a similar increase in fluorescence and this response was abolished in the presence of 30 µM mecamylamine (Fig. 1). The α7-selective antagonist methyllycaconitine (MLA; 100 nM) was without effect. This suggests that under the conditions of the assay, sazetidine-A activates α3-containing nAChRs but not α7 nAChRs, consistent with previous findings for other agonists [24]. However, in the presence of the α7-selective positive allosteric modulator (PAM) PNU-120596 (10 µM) [25], sazetidine-A evoked significantly larger increases in fluorescence that were partially blocked by both mecamylamine and by MLA (Fig. 1). This suggests that PNU-120596 reveals an α7 nAChR-mediated increase in intracellular Ca^2+^.

**Fig. 2:**
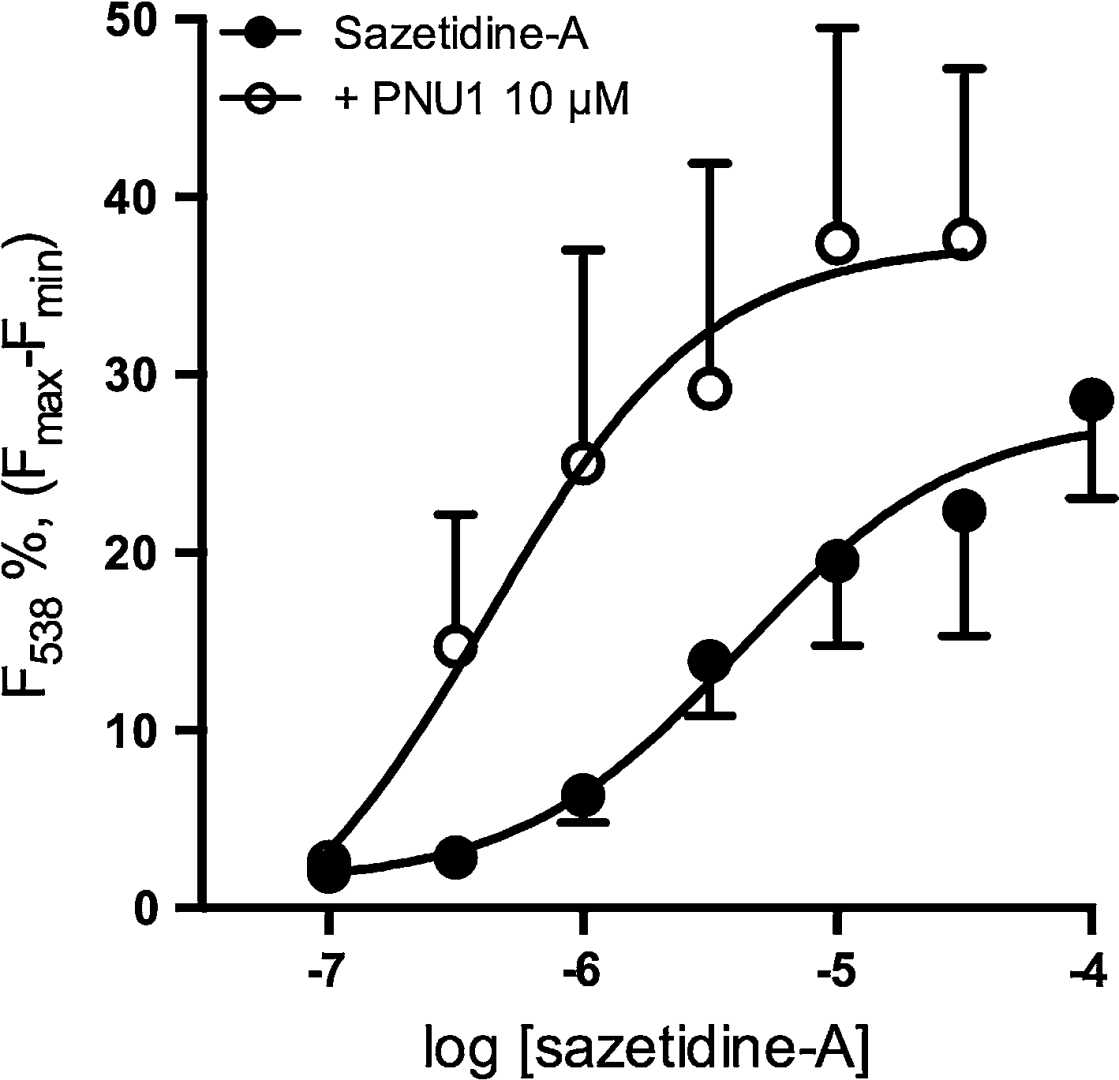
Concentration dependence of sazetidine-A-evoked responses in SH-SY5Y cells. SH-SY5Y cells loaded with fluo-3 AM were stimulated with sazetidine-A (0.1–100 μM) in the presence (*solid black circles*) or absence (*open black circles*) of PNU-120596 (PNU1; 10 μΜ). Fluorescence at 538 nm was measured for 20 s following stimulation with sazetidine-A. The increase in fluorescence at 20 s is presented as a percentage of the maximum fluorescence determined by addition of 0.2 % triton X-100 minus the minimum fluorescence quenched by 350 mM MnCl_2_.* Points* represent the mean ± SEM from 8 independent experiments. Data were fitted to the Hill equation and EC_50_ values for sazatidine-A in the absence and presence of PNU-120596 were calculated to be 4.2 and 0.4 µM respectively

The response elicited by 10 µM sazetidine-A in the presence of PNU-120596 (2.3 ± 0.7 fold increase in fluorescence, Fig. 1) is comparable to that observed with the structurally related agonist 5-iodo-A85380 (1 µM; 2.3 ± 0.2 fold increase) and with nicotine (30 µM; 4.0 ± 0.2 fold increase), both tested in the presence of the PAM (data not shown). Increases in fluorescence in response to sazetidine-A were concentration dependent (Fig. 2). The concentration response curve was shifted to the left in the presence of PNU-120596. EC_50_ values of 4.2 and 0.4 µM were derived for sazetidine-A in the absence and presence of PNU-120596, respectively.

**Fig. 3:**
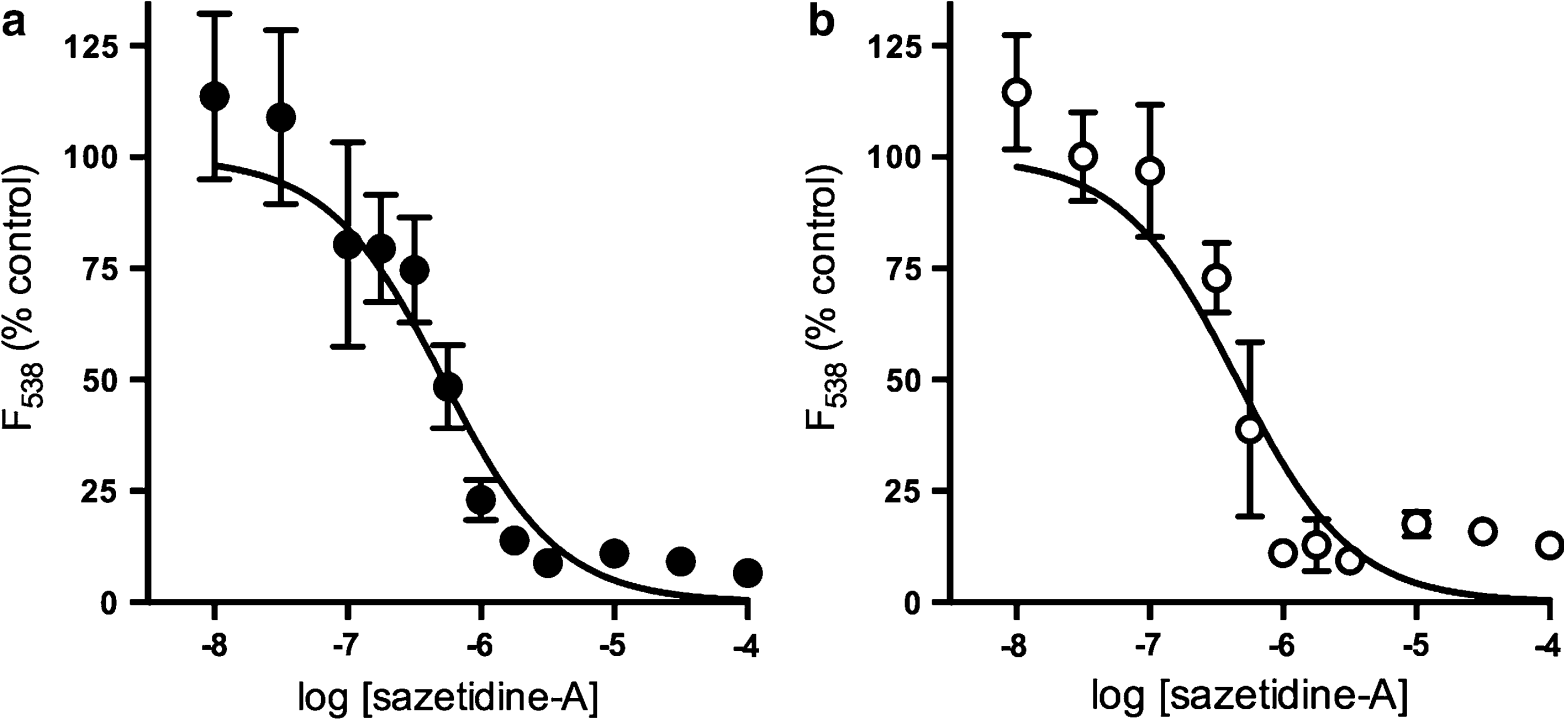
Sazetidine-A inhibits responses evoked by nicotinic agonists in SH-SY5Y cells. SH-SY5Y cells loaded with fluo-3 AM and preincubated with sazetidine-A (0.01–100 μM) for 10 min before stimulation with nicotine (100 μM; *solid circles*, **a**) or PNU-282987 (3 μM) together with PNU-120596 (10 μM; *open circles*, **b**). The PAM was also present during the preincubation period in **b**. Fluorescence at 538 nm was measured for 20 s following stimulation. Normalised responses at 20 s are expressed as a percentage of the response to agonist in the absence of sazetidine-A. Points represent mean ± SEM of at least 4 independent experiments and are fitted to the Hill equation, yielding IC_50_ values of 522 nM and 476 nM for sazetidine-A versus nicotine and versus PNU-282987, respectively

The propensity of sazetidine-A to antagonize nAChRs in SH-SY5Y cells was assessed by preincubating cultures with increasing concentrations of sazetidine-A for 10 min, followed by stimulation with 100 µM nicotine (to activate α3-containing nAChRs) or the α7-selective agonist PNU-282987 (10 µM), in the presence of the PAM PNU-120596. Maximally effective agonist concentrations were deployed to elicit the optimum signal for quantitating inhibition. In both cases sazetidine-A produced a concentration-dependent inhibition of agonist-evoked responses, with IC_50_ values of 522 and 476 nM respectively (Fig. 3).

### Effects of Sazetidine-A on α7 nAChR-Mediated Ca^2+^ Signals in Mouse Cortical Neurons

**Fig. 4:**
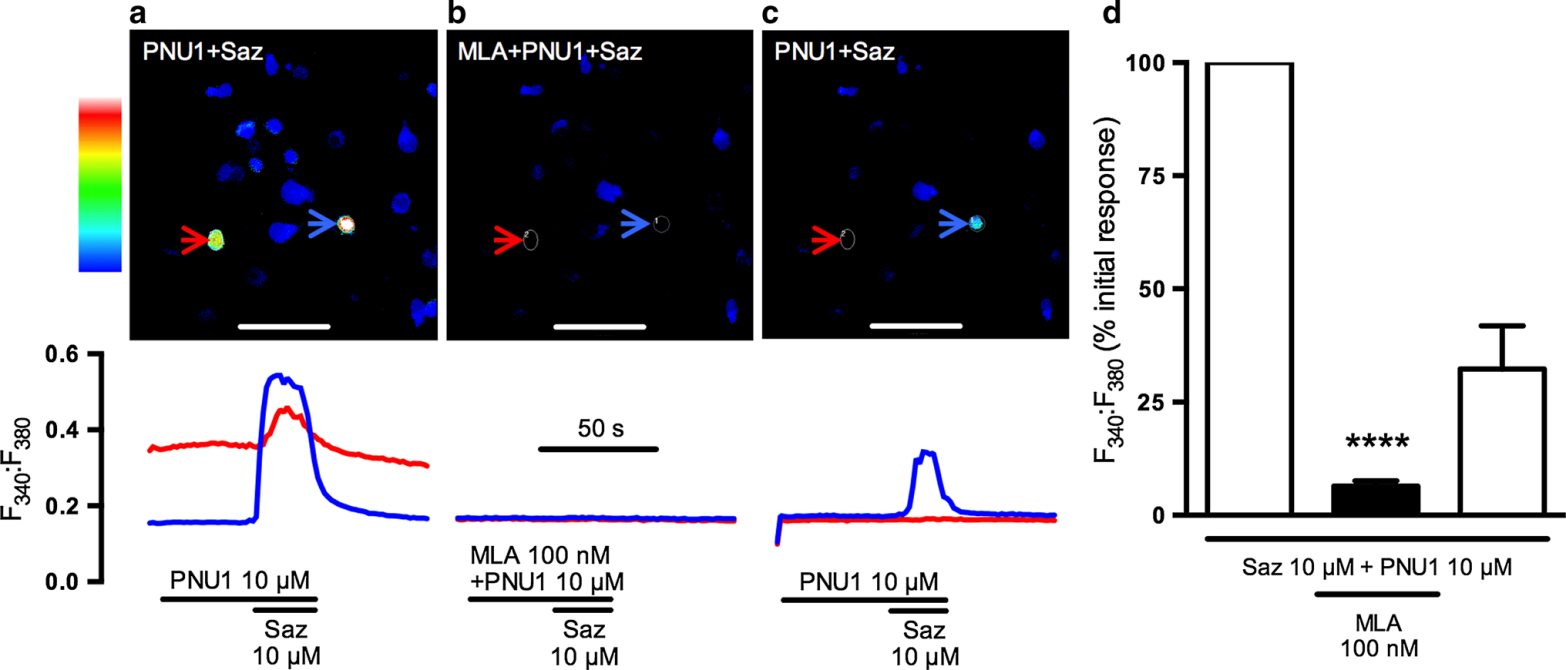
Sazetidine-A elicits intracellular calcium elevations in the presence of PNU-120596 in mouse cortical cultures. Mouse E18 primary cortical cultures (10–14 DIV) were loaded with fura-2 AM, perfused with 1.8 mM calcium buffer at 37 °C and imaged under a fluorescence microscope at 510 nm. Cultures were pre-incubated with PNU-120596 (PNU1; 10 μM; 3 min). Basal fluorescence (F_340_:F_380_) was recorded for 30 s before stimulation with sazetidine-A (Saz; 10 μM; 20 s). Following 3 min washout, cells were pre-incubated with MLA (100 nM; 10 min) and PNU-120596 (10 μM; 3 min) prior to recording F_340_:F_380_ before, during and after co-stimulation with sazetidine-A (10 μM; 20 s). Following 10 min washout, the protocol was repeated in the absence of MLA. *Panels* **a**, **b**, **c** show representative images of the same field of cells taken during the 3 successive stimulations with sazetidine-A before, during and after exposure to MLA; *scale bar* 150 µm. Fluorescence is shown in pseudocolour, (*black/blue* = low F_340_:F_380_, *red/white* = high F_340_:F_380_). Two cells that responded to stimulation in a representative experiment are indicated by the *arrowheads* and their individual fluorescence profiles are shown below; fluorescence changes are presented as a ratio of fluorescence emitted at 510 nm following excitation at 340 and 380 nm. **d** Averaged data from 3 independent cultures are presented graphically. *Bars* represent the mean ± SEM peak F_340_:F_380_ increase above basal, expressed as a percentage of the response to the initial stimulation with sazetidine-A in the presence of PNU-120596, from the same region of interest. ****P < 0.0001 significantly different from initial response to sazetidine-A in combination with PNU-120596, one sample *t*-test

Experiments were carried out on mouse E18 primary cortical cultures to assess the effects of sazetidine-A on native α7 nAChRs in cells with a more neuronal phenotype. Cortical cultures were loaded with fura-2 AM and changes in fluorescence indicative of changes in intracellular Ca^2+^ were monitored by live cell imaging. Sazetidine-A alone (10 nM–10 µM; 20 s application) failed to evoke any change in fluorescence, except for occasional, inconsistent increases at the highest concentration tested. In contrast, 40 mM KCl consistently produced robust responses from a majority of cells (data not shown). Following preincubation with PNU-120596, co-application of 10 µM sazetidine-A with the PAM evoked sustained responses from 14 % of cells (average from 6 experiments from 3 independent cultures). Responses were completely blocked by 100 nM MLA, with partial recovery (32.4 ± 9.4 % of initial response) following 10 min washout (Fig. 4).

**Fig. 5:**
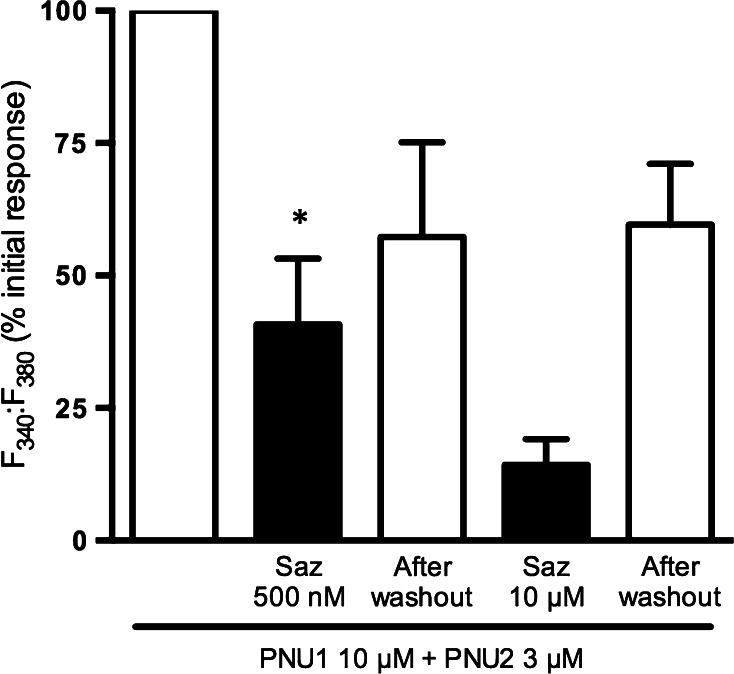
Sazetidine-A attenuates responses to PNU-282987 in cortical cultures. Mouse E18 primary cortical cultures (10–14 DIV) were loaded with fura-2 AM, perfused with 1.8 mM calcium buffer at 37 °C and imaged under a fluorescence microscope at 510 nm. Cultures were pre-incubated with PNU-120596 (PNU1; 10 μM; 10 min). Basal fluorescence (F_340_:F_380_) was recorded for 30 s before during and after stimulation with PNU-282987 (PNU2; 3 μM; 20 s). Following 3 min washout, cells were pre-incubated with sazetidine-A (Saz; 500 nM or 10 µM) and PNU-120596 (10 μM; 10 min) prior to recording F_340_:F_380_ before, during and after stimulation with PNU-282987 (3 μM; 20 s). Following 10 min washout, the protocol was repeated in the absence of sazetidine-A. Responses are presented as a % of the initial response to PNU-282987, after subtraction of basal values. Bars represent the mean ± SEM of data averaged from 3 (500 nM sazetidine-A) or 1 (10 µM sazetidine-A) independent cultures. *P < 0.05 significantly different from initial response to PNU-282987 in combination with PNU-120596, one sample *t*-test

Sazetidine-A was examined for its ability to attenuate responses from α7 nAChRs in cortical neurons by sequential application of the α7 nAChR agonist PNU-282987 alone (in the presence of PNU-120596) and following exposure to sazetidine-A for 10 min (Fig. 5). Sazetidine-A applied at 500 nM, a concentration approximating the IC_50_ value derived from SH-SY5Y cells (Fig. 3), decreased the response to PNU-282987 by 59 %, whereas preincubation with 10 µM sazetidine-A resulted in a stronger block of 86 %. This effect was not due to run-down of responses or exhaustion of the Ca^2+^ indicator as responses recovered to 57 and 60 % of control, respectively, after 10 min washout of sazetidine-A (Fig. 5).

## Discussion

In this study we have exploited the PAM PNU-120596 to reveal activity of native α7 nAChRs [27], in order to examine the actions of sazetidine-A on α7 nAChRs expressed in SH-SY5Y cells and mouse cortical cultures. In the absence of the PAM, sazetidine-A evoked mecamylamine-sensitive increases in fluorescence in SH-SY5Y cells that were insensitive to MLA. The EC_50_ value of 4 µM is consistent with the activation of human α3β4* nAChRs in SH-SY5Y cells; at heterologously expressed α3β4 nAChRs sazetidine-A is a relatively weak agonist, with efficacy ranging from ~0 to 100 % in different studies, presumably reflecting differences in stoichiometry, species and methodology [1, 7, 9].

The lack of α7 nAChR responses in the absence of the PAM is likely to reflect the rapid kinetics of the receptor, as other agonists were previously found to be without effect in this assay [24]. However, sazatidine-A was recently reported to activate rat α7 nAChRs with very low efficacy [14] although another study using a different assay and overexpressed human α7 nAChRs, reported 100 % efficacy [7]. The MLA-sensitive enhancement of responses to sazetidine-A in the presence of the PAM PNU-120596 is indicative of the recruitment of α7 nAChRs. The lower EC_50_ determined in the presence of PNU-120596 is likely to underestimate the true EC_50_ for sazetidine-A at α7 nAChRs as this PAM shifts the agonist concentration–response relationship to the left by approximately 0.8 of a log unit [25]. This suggests that the EC_50_ value for activation of α7 nAChRs in SH-SY5Y cells by sazetidine-A would be in the low µM range, comparable with the recent report that sazetidine-A activated recombinant α7 nAChRs in the absence of a PAM with an EC_50_ value of 1.2 µM, using a sensitive fluorescence assay to measure changes in membrane potential [7]. A higher EC_50_ value of 60 µM was found using two-electrode voltage clamp recording from Xenopus oocytes expressing rat α7 nAChRs [14].The ability of a brief (10 min) incubation with sazetidine-A to ameliorate responses to subsequent stimulation of α3* or α7 nAChRs is consistent with its propensity to desensitize nAChRs. There was a concern that this experiment would be compromised by the requirement for preincubation with the PAM, alongside sazetidine-A, in order to reveal α7 nAChR-evoked responses. Although PNU-120596 prolongs the activation of α7 nAChRs [25], the duration of this effect is relatively short-lived with return to baseline within 5 min [26]. The very similar inhibition curves for nicotine-evoked responses in the absence of the PAM, attributed to α3β4* nAChRs, and for responses evoked by the α7-selective agonist PNU-282987 in the presence of PNU-120596 argues that an inhibitory effect of sazetidine-A is measured in both cases and that α3β4* and α7 nAChRs are similarly sensitive to inhibition by sazetidine-A.

The IC_50_ values for this effect were ~0.5 µM (Fig. 3). This could be relevant to clinical applications of sazetidine-A when therapeutic concentrations may approach these levels [28] (see below). Moreover, Campling et al. [7] recently highlighted ‘smouldering activation’ of nAChRs resulting from the balance within a population of receptors of sustained desensitization versus activation, such that the impact of chronic agonist concentrations will be complex.

The sensitivity of α7 nAChRs to sazetidine-A was reinforced by studies in primary cortical neurons. Interestingly, no changes in fluorescence were detected in response to sazetidine-A in the absence of the PAM. This was surprising as functional α4β2 nAChRs might have been anticipated to be present in cortical neurons. Possible explanations are that they are only present in the LS-α4_3_β2_2_ stoichiometry, or that they are absent at this developmental stage. Although α4β2 nAChRs have been documented on thalamocortical afferents [29], projection neurons would not be present in the cortical cultures. However α4β2 nAChRs may also occur on intrinsic cortical neurons [30, 31]. Alternatively, α4β2 nAChRs might not initiate detectable changes in intracellular Ca^2+^, due to the presence of TTX in the perfusing buffer.

In contrast, in the presence of PNU-120596 sazetidine-A elicited robust responses from a minority of cells, estimated as 14 % of the total population. This proportion is consistent with measurements using a selective α7 nAChR agonist together with the PAM (Brown and Wonnacott, unpublished observation). The almost total blockade of these responses by 100 nM MLA confirmed that they arise from activation of α7 nAChRs. Recovery following 3 min washout was partial and somewhat variable, possibly reflecting sazetidine-A’s propensity to desensitize nAChRs. This was supported by the ability of sazetidine-A to produce a concentration-dependent-decrease in responses to PNU-282987, with sensitivity similar to that observed in SH-SY5Y cells.

Together these data provide evidence for the ability of low micromolar concentrations of sazetidine-A to activate native human and mouse α7 nAChRs, whereas an inhibitory effect, likely reflecting desensitization of α7 nAChRs, was observed at sub-micromolar concentrations of sazetidine-A. Brain concentrations of sazetidine-A administered chronically to rodents via osmotic minipump (4.7 mg/kg/day) have been estimated to reach 32 nM, but repeated injection achieved transient levels that were 10 times higher [28]. This would be sufficient to elicit a degree of desensitization and/or ‘smouldering activation’ of α7 nAChRs [7], which could either compromise or contribute to the beneficial effects of a selective agonist such as sazetidine-A.
